# Severe COVID anxiety among adults in the United Kingdom: cohort study and nested feasibility trial

**DOI:** 10.1186/s12888-023-05446-9

**Published:** 2024-01-06

**Authors:** Mike J. Crawford, Jacob D. King, Aisling McQuaid, Paul Bassett, Verity C. Leeson, Oluwaseun Tella, Martina Di Simplicio, Peter Tyrer, Helen Tyrer, Richard G. Watt, Kirsten Barnicot

**Affiliations:** 1 Division of Psychiatry, Commonwealth Building, Imperial College London, Hammersmith Hospital Campus, Du Cane Road, London, W12 0NN UK; 2 Statsconsultancy Ltd, Amersham, Buckinghamshire UK; 3 Department of Epidemiology & Public Health, University College London, 1-19 Torrington Place, London, WC1E 7HB UK; 4 Division of Health Services Research and Management, City, University of London, Northampton Square, London, EC1V 0HB UK

**Keywords:** COVID-19, Anxiety disorders, Cohort study, Feasibility trial

## Abstract

**Background:**

People with severe COVID anxiety have poor mental health and impaired functioning, but the course of severe COVID anxiety is unknown and the quality of evidence on the acceptability and impact of psychological interventions is low.

**Methods:**

A quantitative cohort study with a nested feasibility trial. Potential participants aged 18 and over, living in the UK with severe COVID anxiety, were recruited online and from primary care services. We examined levels of COVID anxiety in the six months after recruitment, and factors that influenced this, using linear regression. Those scoring above 20 on the short Health Anxiety Inventory were invited to participate in a feasibility trial of remotely delivered Cognitive Behavioural Therapy for Health Anxiety (CBT-HA). Exclusion criteria were recent COVID-19, current self-isolation, or current receipt of psychological treatment. Key outcomes for the feasibility trial were the level of uptake of CBT-HA and the rate of follow-up.

**Results:**

204 (70.2%) of 285 people who took part in the cohort study completed the six month follow-up, for whom levels of COVID anxiety fell from 12.4 at baseline to 6.8 at six months (difference = -5.5, 95% CI = -6.0 to -4.9). Reductions in COVID anxiety were lower among older people, those living with a vulnerable person, those with lower baseline COVID anxiety, and those with higher levels of generalised anxiety and health anxiety at baseline. 36 (90%) of 40 participants enrolled in the nested feasibility trial were followed up at six months. 17 (80.9%) of 21 people in the active arm of the trial received four or more sessions of CBT-HA. We found improved mental health and social functioning among those in the active, but not the control arm of the trial (Mean difference in total score on the Work and Social Adjustment Scale between baseline and follow up, was 9.7 (95% CI = 5.8–13.6) among those in the active, and 1.0 (95% C.I. = -4.6 to 6.6) among those in the control arm of the trial.

**Conclusions:**

While the mental health of people with severe COVID anxiety appears to improve over time, many continue to experience high levels of anxiety and poor social functioning. Health anxiety is highly prevalent among people with severe COVID anxiety and may provide a target for psychological treatment.

**Trial registration:**

Retrospectively registered at ISRCTN14973494 on 09/09/2021.

**Supplementary Information:**

The online version contains supplementary material available at 10.1186/s12888-023-05446-9.

## Introduction

For many people, the COVID pandemic had a negative impact on their mental health [1, 2]. A number of studies have demonstrated marked increases in the proportion of people who experienced anxiety during the early stages of the pandemic, which appeared to be associated with social isolation, economic hardship and concerns about personal health and the health of others [3–5]. For some, fears about contracting the coronavirus and the impact of COVID-19 on society had a negative impact on their mental health [6, 7], People with severe COVID anxiety spent more time online reading about COVID than people with lower levels of anxiety [6] and often took steps to try to avoid the virus that went far beyond recommended public health guidance [8]. People with severe COVID anxiety had poor mental health and social functioning compared to those with lower levels of COVID anxiety [8, 9]. As many as 70% of people with severe COVID anxiety reported other worries about their health and the consequences of becoming unwell [8] and it has been argued that the spread of COVID may have exacerbated the fears of people who already had a tendency to be overly anxious about their health [10].

As the pandemic progressed, variants of the virus emerged that were associated with lower levels of morbidity and mortality. It might be supposed that this, together with the development of improved medical treatments for COVID-19 and the roll out of effective vaccination programmes, would mean that people with severe COVID anxiety would become less anxious. However, to date longitudinal studies of people with severe COVID anxiety have not been conducted, and its course is unclear.

Soon after the start of the pandemic, healthcare services and national and international bodies developed guidance for people whose mental health had been affected by the pandemic. In the absence of evidence from clinical trials, this guidance took the form of general advice about how to look after mental health, such as taking regular exercise and avoiding alcohol or drug misuse [11, 12]. In the absence of evidenced-based interventions for helping people with severe COVID anxiety, we judged that a psychological intervention focussed on health anxiety may provide a more effective response to people who experienced these problems. We based this judgement on: (i) data highlighting the role of health anxiety in poor mental health during previous pandemics, [13, 14] (ii) clinical experience of assessing patients who contacted mental health services with severe COVID anxiety in the context of wider worries about their physical health, and (iii) evidence demonstrating the clinical effectiveness of Cognitive Behavioural Therapy for Health Anxiety (CBT-HA) [15, 16]. Previous research has also shown that CBT-HA can be delivered remotely, [17] an important consideration when planning psychological support for people who may be wanting to limit their face-to-face contacts with others. We therefore designed a study to examine the course of severe COVID anxiety, and to test the acceptability and feasibility of remotely delivered CBT-HA for people who had severe COVID anxiety in the context of wider worries about their physical health.

Our primary aim was, among a group of people who self-identified as having severe COVID anxiety, to examine assess their level of COVID anxiety six months later. Our secondary aims were (1) generate hypotheses about clinical and demographic factors that are associated changes in the level of COVID anxiety, and (2) to test the feasibility of a randomised controlled trial of CBT-HA for people with severe COVID anxiety and health anxiety aimed at improving mental health and social functioning. The target population for the study was people living in the United Kingdom who experienced levels of COVID anxiety that were having a negative effect on their social functioning. We hypothesised that most people with severe COVID anxiety at the start of the study would no longer have severe COVID anxiety six months later and that the level.

## Methods

We conducted a national cohort study and nested feasibility trial among people living in the United Kingdom. We followed up all study participants three and six months after their entry into the study. The nested trial was an individually randomised, parallel-arm, single (researcher) masked feasibility study comparing CBT-HA with treatment as usual. The study methods have been described in detail in a trial protocol paper and are summarised here [18]. Results of the study are reported in accordance with the Consolidated Standards of Reporting Trials (CONSORT) [19, 20]. Recognising that many people with severe COVID anxiety would be unwilling to undertake face-to-face assessments or treatments, we designed the study so that all procedures were conducted remotely.

### Participants

We recruited members of the public in the UK and patients registered with NHS primary care services in London who self-identified as being anxious about COVID-19. Recruitment took place between February 2021 and September 2021. We started recruiting to the study when ‘lockdowns’ were in place across the UK. In February 2021, schools were closed and severe restrictions were placed on travel and meeting people from outside your household. These restrictions were steadily lifted during the recruitment period and by September 2021 all had been lifted.

We recruited members of the public via adverts posted on social media platforms (Facebook, Reddit, Instagram and Twitter) and websites of mental health charities (MQ Research and Anxiety UK). We also publicised the study via text messages sent to patients registered with 19 primary care practices in North West London. Adverts and text messages invited people who were feeling ‘so anxious about COVID that it is stopping them from getting on with their lives’ to visit a website to find more about the study. Potential participants who visited the site were asked to read a Participant Information Sheet and provide electronic consent before being asked to complete a screening questionnaire to find out if they were eligible to take part. Study participants needed to be aged 18 or over, live in the United Kingdom and score nine or more on the Coronavirus Anxiety Scale (CAS) [9]. We used a threshold of nine or more on the CAS because this identifies those with moderate or severe functional impairment [9]. We excluded people who reported having a current or previous diagnosis of a psychotic mental disorder.

We recruited potential participants for the nested feasibility trial from those taking part in the cohort study. Because the intervention we were examining was based on a modified form of Cognitive Behavioural Therapy for Health Anxiety, we restricted participation in the feasibility trial to people who, in addition to having severe COVID anxiety, also had a clinically significant level of health anxiety. To take part in the feasibility trial, a member of the cohort study had to have a score of 20 or more on the Short Health Anxiety Inventory (sHAI) [21]. We also excluded people from the feasibility trial if, at the time of the baseline assessment, they had had COVID-19 in the previous four weeks, were self-isolating on the advice of a doctor or the NHS Test and Trace service, or if they were already receiving psychological treatment for any mental health condition.

### Procedures

**Fig. 1:**
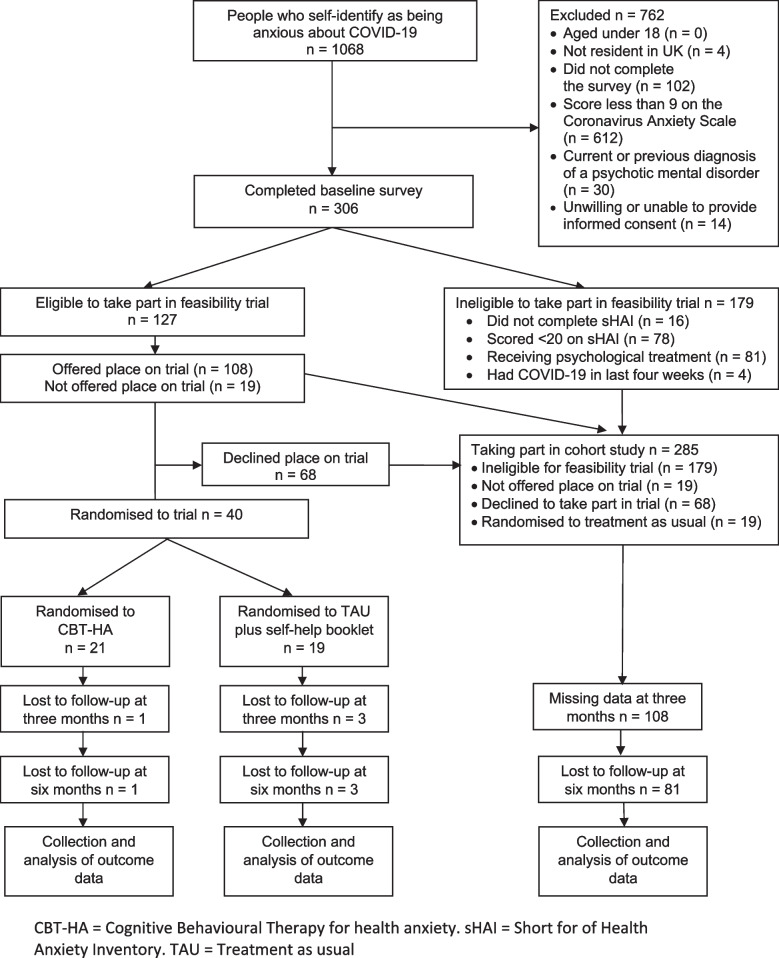
CONSORT flow diagram

Participant flow through the study is presented in Fig. 1. Study data were collected via a website hosted by Qualtrics (www.qualtrics.com). We designed study surveys so that they could be completed only once from any one Internet Protocol address [22]. Potential participants who met eligibility criteria and provided informed consent were invited to complete a baseline survey. Those who were ineligible were directed to a webpage which listed sources of mental health support. We identified potential participants for the feasibility trial based on their responses to the baseline survey. We had to adjust recruitment to the feasibility trial according to the capacity of study therapists to take on new participants. When therapists had capacity to work with a new participant, we emailed those who met the additional eligibility criteria for the trial with a copy of an additional Participant Information Sheet and directed them to a second online consent form. A researcher then contacted these participants by telephone to answer any queries they had about the study, confirm that they met study eligibility criteria, support them to complete the online consent form and counter sign it. Any participant who was approached to take part in the randomised trial and was ineligible or declined to take part, remained in the cohort study and was asked to complete subsequent follow-up surveys. We included a trick questions [22, 23] in an effort to prevent responses from automated responders or ‘bots’. The trap question provided a range of valid and non-valid but plausible options for how the potential participant had heard about the study, with only those providing a valid response being invited to complete the baseline survey.

All participants in the cohort study and the feasibility trial were sent an automated email to ask them to complete the three- and six-month follow-up survey. Those who did not respond after one week were sent email reminders from a researcher. Trial participants who did not complete follow-up interviews were also contacted by telephone to ask them to complete the follow-up surveys.

### Randomisation and masking

Consecutive cohort study participants who were eligible to take part in the feasibility trial were randomised to CBT-HA or treatment as usual in a 1:1 ratio. Allocation codes were generated using the independent web-based 'sealed envelope' (https://www.sealedenvelope.com/simple-randomiser/v1/lists) service. We stratified randomisation according to total scores on the short Health Anxiety Inventory (< 24 and 24 and above) and the Dependent Personality Questionnaire (< 11 and 11 and above). We included the latter because of evidence that it can influence uptake and outcomes of the intervention we were testing [24]. The trial manager allocated the participant, according to the randomisation list and notified the participant and the therapist for those allocated to CBT-HA. We minimised measurement bias by selecting outcome measures which participants completed online without assistance from researchers, and by limiting contact that unmasked members of the study team had with participants following randomisation.

### Assessments

#### Choice of primary measure

Our primary outcome was COVID anxiety, measured using the Coronavirus Anxiety Scale (CAS) [9, 25]. Developed by Sherman Lee in the USA during the first wave of the COVID-19 pandemic, this scale was designed to provide a brief and reliable measure of the level of COVID-related anxiety. It consists of five questions on the frequency of anxious thoughts, somatic symptoms and sleep disturbance triggered by thinking, hearing or reading, about COVID. We selected the CAS as our primary outcome measure because it was the only validated measure of COVID anxiety when we developed the study protocol, and it is the most widely used measure of COVID anxiety. A score of nine or more on the CAS indicates severe COVID anxiety, [9, 26]. We used change in score on the CAS for our analysis of factors that were associated with changes in level of COVID anxiety over the six month period.

#### Baseline assessment and covariates

We collected self-reported data on demographic factors including age, gender, ethnicity, household composition, occupational status, physical health and exposure to COVID (whether the participant had had COVID, and whether they had been admitted to hospital with COVID). We asked participants whether they live with or care for someone that might get seriously ill if infected with COVID and whether someone in their family or a close friend had ever been admitted to hospital with COVID. We asked participants about behaviours intended to reduce the risk of exposure to COVID. These questions were developed with the help of the members of our Lived Experience Advisory Panel and drew on what they had done to try to avoid contracting COVID: staying at home, avoiding shops, washing or discarding letters and parcels, increased handwashing and increased washing of clothes. People who lived with school age children were also asked whether they had stopped them attending school because of their concerns about COVID.

We assessed self-reported functioning using the Work and Social Adjustment scale (WSAS) [27] and assessed mental health using the Patient Health Questionnaire-9 (PHQ-9), [28], Generalised Anxiety Disorder 7-item scale (GAD-7), [29]. Obsessive Compulsive Inventory-Revised (OCI-R), [30] the short form of the Health Anxiety Inventory (sHAI), [21] the Standardised Assessment of Personality Abbreviated Scale (SAPAS), [31] and the Dependent Personality Questionnaire (DPQ) [32]. We assessed alcohol use using the Alcohol Use Identification Test – Consumption (AUDIT-C) [33] and use of illicit drugs using a single screening question [34].

#### Follow-up surveys

All those taking part in the study were asked to complete follow-up surveys three and six months after the baseline survey. The content of these surveys was the same as that at baseline, except we did not repeat the personality assessments, and we added a question at six months on whether people had received a COVID-19 vaccination.

We recorded data on Serious Adverse Events (SAE) including death, hospitalisation and life-threatening events at baseline, and three and six month follow-up. Therapists were also reminded that they should report all SAEs to the clinical trial team. All those who completed a baseline interview were offered a £10 gift voucher and all those who completed the six-month follow-up interview were offered an additional £20 voucher.

### Interventions

All participants were sent a self-help booklet giving general advice on maintaining good mental health and wellbeing during the pandemic that was developed by staff at Central and North West London NHS Foundation Trust [35]. They were also able access treatment as usual, such as NHS primary care and referral on to secondary care services if required. In addition to this, all participants in the active arm of the trial were offered five to ten sessions of Cognitive Behavioural Therapy for Health Anxiety (CBT-HA) based on a published treatment manual [36]. Therapists started by taking a detailed history of the person’s thoughts and fears about COVID and exploring their beliefs about the possible impact of COVID infection. Therapists used Beck’s Anxiety Equation to explore participants’ beliefs about their ability to cope with getting COVID. They also sought to identify behaviours that might be maintaining their anxiety, such as searching the internet for information about COVID and its consequences, seeking reassurance, and monitoring bodily symptoms for signs that they could have COVID. Therapists then used diary keeping, Socratic dialogue, and behavioural experiments to help people try to make links between their thoughts, behaviour and their mental health, and explore ways that they might be able to reduce their anxiety.

All CBT-HA sessions were delivered by videoconferencing software on a weekly or fortnightly basis and lasted between 30 and 50 min. Therapists usually delayed their final session to give people an opportunity to use the techniques and skills they had learned and reinforce the changes they had made. The content of sessions was modified to meet the difficulties that people in the study experienced. This included using graded exposure to help people begin to re-engage with activities they had curtailed or stopped since the start of the pandemic. Sessions were supplemented by a booklet summarising the causes of health anxiety and steps that people with health anxiety can take to improve their mental health. Each therapist recorded the number, duration and content of the sessions they delivered.

All therapists had a degree in a health-related subject and had previous experience of delivering psychological treatments. They received a 90-min training session followed by fortnightly supervision sessions, which were delivered by Dr Helen Tyrer (an expert in the treatment of people with health anxiety).

### Statistical analysis

We published a statistical analysis plan prior to the start of data analysis (https://www.isrctn.com). We aimed to recruit sufficient numbers of participants to the cohort study to randomise 40 participants to the feasibility trial; a typical size for such a trial [37]. A sample of 40 participants was sufficient to enable us to detect a 50% uptake of CBT-HA, with 95% confidence intervals of ± 15%.

The study cohort comprised all participants with the exception of those offered CBT-HA as part of the feasibility trial. We excluded these participants because we hypothesised that the psychological support they received could have an impact on their recovery.

For the cohort study, we started by comparing demographic and clinical factors among those who did and did not complete the six month follow up survey. We then examined changes in CAS score at three and six months after completion of the baseline survey.

We conducted a complete case analysis to examine factors that influenced changes in CAS scores over the six-month follow-up period using linear regression analysis. The analysis was performed in two stages. Initially the association between each factor and CAS at six months was assessed separately. We adjusted for CAS score at baseline in all these analyses, so that the analyses reflected factors associated with change in CAS from baseline. The second stage of the analysis involved examining the joint association between the factors and CAS score in a multivariable model. To restrict the number of variables in this stage of the analysis, only factors showing some association with the outcome (p < 0.2) from the first stage of the analysis were included. We used backwards selection to identify factors significantly associated with the total CAS score at six months.

The criteria for determining the success of the feasibility study, were based on thresholds used in other feasibility and pilot trials, [38, 39] recruitment of at least 32 participants (80% of the target study sample of 40 participants), uptake of the intervention by at least 60% of participants in the active arm of the trial, and completion of follow-up interviews at six months by 75% of study participants. All data were analysed in SPSS version 20.0 (SPSS Inc., Chicago IL) and Stata version 16.1 [40].

## Results

Of 1,068 potential participants, 966 completed the screening survey. 48 (5.0%) people who completed the screening survey were excluded because they did not provide consent, lived outside the UK or had a history of psychosis. A further 612 (63.4%) scored less than 9 on the CAS and were also therefore excluded (see Fig. 1).

Detailed information about the 306 people who took part in the study are published elsewhere [8]. 246 (81.2%) were female, the median age was 41 (range 18–83), and 210 (70.2%) of the 299 that indicated their ethnicity is White British or Irish. A total of 139 (46.0%) reported being employed, 47 (15.6%) unemployed and 39 (12.9%) were students with the remainder retired, carers or “furloughed” (receiving a proportion of their salary from the UK government, but not actively working). 192 (60.3%) of the sample reported a mental or physical health condition. The most commonly reported were anxiety (62; 21.2%), asthma (40; 13.7%), depression (36; 12.3%), hypertension (26; 8.9%), fibromyalgia (18; 6.2%), diabetes mellitus (18; 6.2%), irritable bowel syndrome (13; 4.5%), and non-inflammatory arthritis (13; 4.5%). One quarter (76; 26.0%) of the sample reported having a condition that was associated with an increased risk of hospitalisation and mortality from COVID-19 [41]. 20 (6.5%) participants were recruited from primary care practices with the remainder recruited from websites of voluntary sector organisations and social media.

Two hundred and eight five people took part in the cohort study; 306 participants minus 21 who were in the active arm of the feasibility trial. Of these, 177 (62.1%) completed the three-month follow-up survey and 204 (71.6%) completed the six-month survey. People who did not take part in the six-month follow-up study were more likely to be employed, have better social functioning and be living with a person who was vulnerable to the effects of COVID (see Table 1).

**Table 1:** Baseline demographic and clinical characteristics

Variable	Category	Completers (*N* = 204)		Non-completers (*N* = 81)	
		**n**	**Summary**	**n**	**Summary**
Age	Age	201	41.2 [28.5, 53.1]	81	35.3 [27.5, 52.1]
Gender	Female	201	162 (80.6%)	81	64 (79.0%)
	Male		39 (19.4%)		17 (21.0%)
Ethnicity	White	198	162 (81.8%)	78	61 (78.2%)
	Asian		21 (10.6%)		5 (6.4%)
	Black		9 (4.6%)		3 (3.9%)
	Mixed		4 (2.0%)		7 (9.0%)
	Other		2 (1.0%)		2 (2.6%)
In employment	No	201	118 (58.7%)	80	35 (43.7%)
	Yes		83 (41.3%)		45 (56.3%)
Lives alone	No	201	166 (82.6%)	80	64 (80.0%)
	Yes		35 (17.4%)		16 (20.0%)
Lives with a	No	198	128 (64.6%)	78	38 (47.7%)
vulnerable person	Yes		70 (35.4%)		40 (51.3%)
Medical	No	200	72 (36.0%)	80	35 (43.7%)
condition	Yes		128 (64.0%)		45 (56.3%)
At risk	No	200	144 (72.0%)	80	66 (82.5%)
condition	Yes		56 (28.0%)		14 (17.5%)
Drug use in the	No	193	178 (92.2%)	71	62 (87.3%)
last year	Yes		15 (7.8%)		9 (12.7%)
CAS	-	204	12.2 ± 2.7	81	12.8 ± 3.6
WSAS	-	195	22.0 ± 7.4	72	19.3 ± 8.1
GAD-7	-	197	16 [13, 19]	77	16 [12, 19]
sHAI	-	196	23.3 ± 6.7	73	23.0 ± 8.3
PHQ-9	-	196	15.5 ± 5.5	72	15.8 ± 5.6
OCI-R	-	193	29.9 ± 14.8	71	31.7 ± 16.6
SAPAS	-	192	4.2 ± 1.8	71	4.0 ± 1.9
DPQ	-	193	10.6 ± 4.1	71	11.7 ± 4.1
AUDIT-C	-	193	2 [0, 4]	71	3 [0, 6]

Summary statistics are: mean ± standard deviation, median [inter-quartile range], or number (percentage)

**Fig. 2:**
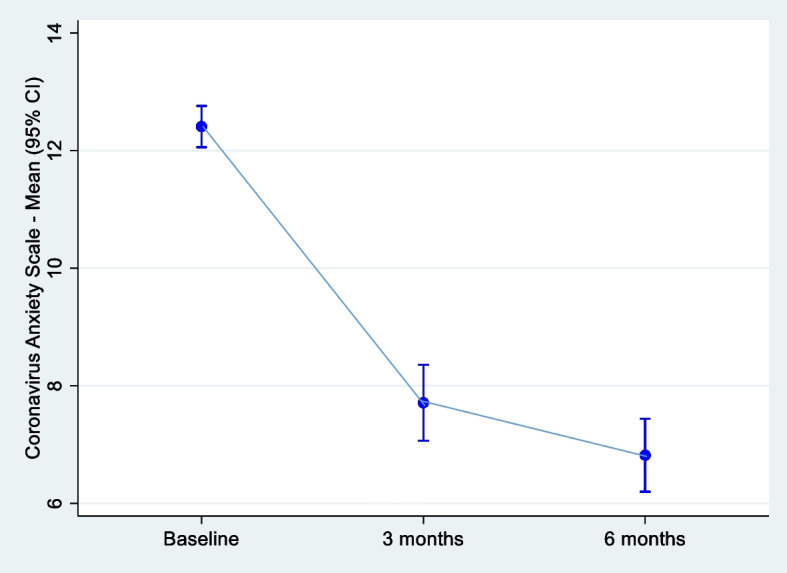
Mean scores on the Coronavirus Anxiety Scale values over six months

Changes in total CAS score between these three time points are presented in Fig. 2.

Clinically and statistically significant reductions in social dysfunction, generalised anxiety and most other measures of mental health were also seen during this period (see Table 2). Changes in COVID-related thoughts and behaviours over time are presented in Table 3 (below).

**Table 2:** Changes in mental health and social functioning over six months

Variable	Timepoint	n	Mean ± SD	Change Mean (95% CI)	*p*-value vs. baseline
CAS	Baseline	285	12.4 ± 3.0	0	
	3 months	177	7.7 ± 4.4	-4.7 (-5.2, -4.1)	** < 0.001**
	6 months	204	6.8 ± 4.5	-5.5 (-6.0, -4.9)	** < 0.001**
WSAS	Baseline	267	21.3 ± 7.7	0	
	3 months	169	19.0 ± 9.0	-2.7 (-3.9, -1.5)	** < 0.001**
	6 months	203	18.3 ± 9.0	-3.4 (-4.5, -2.3)	** < 0.001**
GAD-7	Baseline	274	15.4 ± 4.0	0	
	3 months	172	13.4 ± 5.0	-2.2 (-2.9, -1.5)	** < 0.001**
	6 months	203	12.5 ± 5.5	-3.0 (-3.7, -2.3)	** < 0.001**
sHAI	Baseline	269	23.2 ± 7.1	0	
	3 months	171	21.7 ± 8.5	-1.8 (-2.6, -0.9)	** < 0.001**
	6 months	203	19.7 ± 8.4	-3.5 (-4.3, -2.7)	** < 0.001**
PHQ-9	Baseline	268	15.6 ± 5.5	0	
	3 months	170	13.8 ± 6.4	-1.5 (-2.3, -0.7)	** < 0.001**
	6 months	203	13.4 ± 6.7	-2.1 (-2.9, -1.4)	** < 0.001**
OCI-R	Baseline	264	30.4 ± 15.3	0	
	3 months	169	28.2 ± 16.8	-1.5 (-3.2, 0.2)	0.09
	6 months	203	25.9 ± 15.5	-4.1 (-5.7, -2.5)	** < 0.001**
AUDIT-C	Baseline	264	2.66 ± 2.97	0	
	3 months	173	2.48 ± 2.77	0.03 (-0.22, 0.28)	0.81
	6 months	204	2.41 ± 2.62	0.02 (-0.22, 0.25)	0.88
Variable	Timepoint	n	n (%)	Odds Ratio (95% CI)	*p*-value vs. baseline
Drug use	Baseline	264	24 (9.1%)	1	
	3 months	173	12 (6.9%)	0.61 (0.18, 2.04)	0.42
	6 months	204	20 (9.8%)	1.62 (0.58, 4.52)	0.35

**Table 3:** Changes in COVID-related thoughts and behaviours over six months

Variable	Timepoint	n	n (%)	Odds Ratio (95% CI)	*p*-value vs. baseline
Constant state of worry about COVID	Baseline	268	21.2	1	
	3 months	168	14.3	0.62 (0.37 – 1.04)	0.07
	6 months	203	12.8	0.54 (0.33 – 0.90)	0.02
Constantly	Baseline	268	11.6	1	
watching the	3 months	168	6.5	0.54 (0.26 – 1.10)	0.09
news	6 months	203	4.4	0.35 (0.16 – 0.76)	0.09
Never leaving home	Baseline	268	11.6	1	
	3 months	168	8.3	0.70 (0.36 – 1.35)	0.28
	6 months	203	9.4	0.79 (0.43 – 1.44)	0.44
Not sending children to school	Baseline	94	20.2	1	
	3 months	53	18.9	0.92 (0.39 – 2.15)	0.84
	6 months	66	10.6	0.46 (0.19 – 1.19)	0.11
Buying all food online	Baseline	268	37.3	1	
	3 months	168	36.3	0.96 (0.64 – 1.43)	0.83
	6 months	203	26.1	0.59 (0.40 – 0.88)	0.01
Constantly washing hands	Baseline	268	20.1	1	
	3 months	168	24.4	1.28 (0.81 – 2.03)	0.30
	6 months	203	15.3	0.71 (0.44 – 1.16)	0.17
Washing all food, letters and parcels coming into the home	Baseline	268	31.0	1	
	3 months	168	23.2	0.67 (0.43 – 1.04)	0.08
	6 months	203	18.2	0.50 (0.34 – 0.77)	0.002
Washing clothes every time they are worn	Baseline	268	17.5	1	
	3 months	168	17.3	0.98 (0.59 – 1.63)	0.94
	6 months	203	13.3	0.72 (0.43 – 1.20)	0.21

Univariate associations between exposure variables and changes in CAS score are presented in an Additional file 1. The results of the multivariate analysis of factors associated with changes in total CAS score between baseline and six months are presented in Table 4.

**Table 4:** Multivariable associations with changes in CAS from baseline to 6 months

Factor	Category	Coefficient (95% CI)	*p*-value
Age ^(b)^	-	-0.4 (-0.7, -0.1)	0.02
Lives with vulnerable	No	0	0.04
person	Yes	-1.1 (-2.2, -0.1)	
CAS baseline	-	0.6 (0.4, 0.8)	< 0.001
GAD-7 baseline	Linear term	1.0 (0.1, 1.8)	0.007
	Squared term	-0.04 (-0.07, -0.01)	
sHAI baseline ^(a)^	-	-0.7 (-1.1, -0.3)	0.001
GAD-7 reduction^c^	-	0.2 (0.1, 0.3)	0.002
sHAI reduction ^(a)c^	-	0.6 (0.1, 1.0)	0.02
PHQ-9 reduction ^(a)c^	Linear term	0.1 (-0.5, 0.7)	0.01
	Squared term	0.3 (0.1, 0.5)	

(^a^) Regression coefficients reported for a 5-unit increase in variable

(^b^) Regression coefficients reported for a 10-year increase in age

(^c^) Reduction in these scores refers to the change in scores on these measures from baseline to the 6-month follow-up interview

Older age, living with a vulnerable person, lower baseline CAS, and higher GAD-7 and sHAI were all associated with smaller reductions in CAS score from baseline to 6 months. Greater reductions in sHAI and PHQ-9 were significantly associated with greater reductions in CAS score between baseline and 6 months. The strongest predictor of reduced CAS score was the baseline CAS score (people with higher baseline CAS tended to have the greater reduction in scores at six months). We did not find an association between vaccination status and changes in the level of COVD anxiety (adjusted change in mean CAS score among those who became vaccinated = -0.2, 95% CI = -2.4 to 2.0, p = 0.88).

### Feasibility trial results

Of the 306 study participants, 201 (69.3%, 95% CI = 64.0 to 74.6%) scored 20 or more on the Short Health Anxiety Inventory and 127 (41.5%) met all inclusion criteria for the feasibility trial. Of these, 108 were offered a place in the feasibility trial and 40 (37.0%) accepted and were randomised (21 to CBT-HA and 19 to treatment as usual).

Among the 21 participants in the active arm of the trial, the mean attendance at CBT-HA sessions was 6.7 sessions; 19 (90.5%) attended one or more session and 17 (81.0%) attended four or more sessions. Sessions generally lasted 50 min and took place on a weekly or fortnightly basis. The most frequently delivered elements of CBT-HA were record keeping (100%), steps to reduce symptom monitoring (88%), and use of Beck’s Anxiety Equation (65%) [36]. In addition to this, most patients used graded exposure to help them re-engage with social, occupational and other activities.

Six months after randomisation 36 (90.0%) participants completed a follow-up interview (20, 95.2% in the active arm and 16, 84.2% in the control arm of the trial). Baseline and follow-up scores are presented in Table 5. At six months, 17 (85%) people in the active arm and 12 (75%) of people in the control arm of the trial no longer had severe COVID anxiety. We found evidence of statistically significantly improved mental health on all measured parameters among those in the active arm of the trial. In contrast, the only statistically significant improvement among those in the control arm of the trial was in the level of COVID anxiety.

**Table 5:** Mental health and social functioning among trial participants over six months

Measure	Allocation arm	Baseline mean	3 months	6 months	Mean difference baseline to 6 months [95% CI]
**Coronavirus Anxiety Scale**	Intervention	12.05	7.47	5.05	7.0 [4.6, 9.4]
	Control	12.32	8.87	6.38	5.94 [3.3, 8.6]
**Depression (PHQ-9)**	Intervention	17.05	9.59	9.20	7.85 [5.1, 10.6]
	Control	14.26	12.67	12.94	1.33 [-3.2, 5.9]
**Anxiety (GAD-7)**	Intervention	16.57	11.53	9.55	7.02 [4.4, 9.6]
	Control	15.84	13.53	12.44	3.40 [-0.5, 7.3]
**Health anxiety (sHAI)**	Intervention	27.33	19.94	16.50	10.83 [7.6, 14.1]
	Control	27.63	26.67	23.10	4.57 [-0.1, 9.2]
**Social functioning (WSAS)**	Intervention	20.43	14.0	10.70	9.7 [5.8, 13.6]
	Control	21.95	20.20	20.94	1.01 [-4.6, 6.6]

## Discussion

The results of this study provide information about the course of COVID anxiety among people in the UK. Over the course of a six-month period, mean scores on the Coronavirus Anxiety Scale fell by over 40%. Reductions in COVID-related anxiety were associated with other improvements in mental health during this period including reduced levels of generalised anxiety, health anxiety and depression. Nonetheless, most people continued to report high levels of anxiety about at a level, which is associated with significant social dysfunction. At six-month follow-up, by which time all social restrictions in the United Kingdom had been lifted, a quarter of participants were still buying all their food online and more than one in 20 reported never leaving their home. The mean score on the Work and Social Adjustment Scale at six months remained in the moderate-severe range of the scale [27].

Our second aim was to identify clinical and demographic factors that were associated with changes in the level of COVID anxiety during this period. We found that reductions in COVID anxiety were greater among people who were younger and those with higher CAS scores at baseline. Our results also highlight the importance of social factors (whether someone was living with a person who was vulnerable to the effects of COVID), and mental health (levels of generalised anxiety and changes in levels of anxiety and depression). Our finding that people with lower levels of baseline health anxiety had greater reductions in COVID anxiety, supports the rationale for the nested feasibility trial that we conducted.

Our third aim was to test the feasibility of a randomised controlled trial of CBT-HA for people with severe COVID anxiety and health anxiety aimed at improving mental health and social functioning. Levels of recruitment, uptake of CBT-HA and follow-up met our predefined progression criteria for a larger scale study. In keeping with previous trials of CBT for Health Anxiety, we found evidence that the intervention reduces levels of health anxiety, depression and generalised anxiety [16].

Our study has several limitations. Firstly, the sample was self-selected based on adverts posted on websites and social media and text messages sent to people registered with GPs. This strategy enabled us to recruit our target sample size from around the UK. However, we do not know whether our sample is representative of all people with severe COVID anxiety in the country and our reliance on online recruitment means that we did not recruit people who did not have access to the internet. Secondly, all data were collected from participants after the start of the COVID pandemic. While we have been able to examine the influence of mental health and other factors on the course COVID anxiety, we are not able to examine the impact that these factors had in the aetiology of severe COVID anxiety. It is possible that for many people, the emergence of severe COVID anxiety was a manifestation of a pre-existing mental health issue. Thirdly, 80% of our study sample were women. While this is in keeping with other research that women had higher levels of anxiety about COVID than men, we cannot be sure that our findings are generalisable to men who had severe COVID anxiety. It is possible that other factors that we did not assess, such as socio-economic status, also influenced changes in levels of COVID anxiety during this period. Fourthly, while we selected widely used, validated measures of mental health and social functioning, there were no validated measures of COVID-related behaviours, when we started the study. Since the start of the pandemic other measures have been developed based on a broader definition of COVID anxiety which includes some of these behaviours [42, 43]. While the questions we asked participants were developed with patient involvement, we have not examined their psychometric properties. Fifthly, our nested randomised trial was conducted among people with both severe COVID anxiety and health anxiety. While most people in the cohort study met this criterion, we did not examine interventions for the minority who had severe COVID anxiety but did not also have health anxiety. Finally, we only followed-up study participants for six months and have not examined the longer-term course of severe COVID anxiety. Longer-term follow-up studies are needed.

While we are not aware of any studies which have explored the feasibility of offering CBT-HA to people with severe COVID anxiety, other studies have examined the impact of interventions for anxiety among people who are anxious about COVID. Controlled trials of breathing and relaxation exercises, [44] progressive muscle relaxation, [45] video-based CBT [46] and a self-guided program based on CBT [47] have all reported greater short-term reductions in COVID anxiety among those offered active interventions compared to wait list control. Larger scale trials CBT-HA are needed before we could recommend this approach to helping people with severe COVID anxiety, but the results of the feasibility trial highlight the potential that this approach has for improving the mental health and social functioning of people with severe COVID anxiety who also have co-existing health anxiety.

## Conclusions

The results of this study demonstrate that among people in the UK who self-identified has having COVID anxiety during the pandemic and use social media, levels of anxiety reduced and mental health improved over time. However, a substantial minority of people continue to experience high levels of anxiety and poor social functioning. Health anxiety is highly prevalent among people with severe COVID anxiety and may provide a target for psychological treatment.

### Supplementary Information

**Additional file 1.** Univariable associations with reduction in CAS score from Baseline to 6 months: Demographic factors.

## Data Availability

The data used during the current study are available from the corresponding author on reasonable request.

## Abbreviations

95% CI: 95% Confidence Intervals
AUDIT-C: Alcohol Use Identification Test – Consumption
CAS: Coronavirus Anxiety Scale
CBT-HA: Cognitive Behavioural Therapy for Health Anxiety
CONSORT: Consolidated Standards of Reporting Trials
DPQ: Dependent Personality Questionnaire
GAD-7: Generalised Anxiety Disorder 7-item scale
ISRCTN: International Standard Randomised Controlled Trial Number
OCI-R: Obsessive Compulsive Inventory-Revised
PHQ-9: Patient Health Questionnaire-9
SAE: Serious Adverse Events
SAPAS: Standardised Assessment of Personality Abbreviated Scale
sHAI: Short Health Anxiety Inventory
UK: United Kingdom
WSAS: Work and Social Adjustment scale
