# Supplementary material for: Severe COVID anxiety among adults in the United Kingdom: cohort study and nested feasibility trial
Source: BMC Psychiatry. 2024 Jan 6;24:27. doi: 10.1186/s12888-023-05446-9 (PMC10771646; doi:10.1186/s12888-023-05446-9)
Supplement: Supplementary file 1 — Additional file 1. Univariable associations with reduction in CAS score from Baseline to 6 months: Demographic factors. [file 12888_2023_5446_MOESM1_ESM.docx]

*Additional file 1. Univariable associations with reduction in CAS score from Baseline to 6 months: Demographic factors*

| Factor | Category | CAS Reduction | Unadjusted CAS Baseline | | Adjusted CAS Baseline | |
| --- | --- | --- | --- | --- | --- | --- |
|  |  | Mean ± SD | Coeff (95% CI) | P-value | Coeff (95% CI) | P-value |
| Age ^(**)^ | - | - | -0.7 (-1.1, -0.3) | **<0.001** | -0.7 (-1.1, -0.3) | **<0.001** |
|  |  |  |  |  |  |  |
| Sex | Female | 5.0 ± 4.6 | 0 | **0.009** | 0 | 0.13 |
|  | Male | 7.1 ± 3.7 | 2.1 (0.5, 3.6) |  | 1.2 (-0.4, 2.8) |  |
|  |  |  |  |  |  |  |
| Ethnicity | Not at risk | 5.5 ± 4.6 | 0 | 0.94 | 0 | 0.43 |
|  | At risk | 5.4 ± 4.0 | -0.1 (-1.7, 1.6) |  | -0.6 (-2.3, 1.0) |  |
|  |  |  |  |  |  |  |
| Employment | Not employed | 5.3 ± 4.4 | 0 | 0.57 | 0 | 0.63 |
|  | Employed | 5.7 ± 4.6 | 0.4 (-0.9, 1.6) |  | 0.3 (-0.9, 1.5) |  |
|  |  |  |  |  |  |  |
| Lives alone | No | 5.5 ± 4.5 | 0 | 0.86 | 0 | 0.77 |
|  | Yes | 5.3 ± 4.6 | -0.1 (-1.8, 1.5) |  | -0.2 (-1.8, 1.3) |  |
|  |  |  |  |  |  |  |
| Lives with vulnerable | No | 5.7 ± 4.5 | 0 | 0.20 | 0 | **0.02** |
| person | Yes | 4.9 ± 4.5 | -0.9 (-2.2, 0.5) |  | -1.5 (-2.8, -0.3) |  |
|  |  |  |  |  |  |  |
| Medical condition | No | 5.5 ± 5.1 | 0 | 0.78 | 0 | 0.80 |
|  | Yes | 5.3 ± 4.1 | -0.2 (-1.5, 1.1) |  | 0.2 (-1.1, 1.4) |  |
|  |  |  |  |  |  |  |
| At risk condition | No | 5.5 ± 4.7 | 0 | 0.63 | 0 | 0.57 |
|  | Yes | 5.2 ± 4.0 | -0.3 (-1.7, 1.1) |  | -0.4 (-1.7, 1.0) |  |
|  |  |  |  |  |  |  |
| Drug use in last year | No | 5.5 ± 4.4 | 0 | 0.20 | 0 | 0.25 |
| (Baseline measurement) | Yes | 7.0 ± 3.3 | 1.5 (-0.8, 3.8) |  | 1.3 (-0.9, 3.5) |  |

(**) Regression coefficients reported for a 10-year increase in age

*Univariable associations with changes in CAS score from baseline to 6 months – Clinical factors*

| Baseline score | Category / Term | Unadjusted CAS Baseline | | Adjusted CAS Baseline | |
| --- | --- | --- | --- | --- | --- |
|  |  | Coeff (95% CI) | P-value | Coeff (95% CI) | P-value |
|  |  |  |  |  |  |
| CAS | - | 0.5 (0.3, 0.7) | **<0.001** | - | - |
|  |  |  |  |  |  |
| SAPAS | < 4 | 0 | 0.59 | 0 | 0.76 |
|  | ≥ 4 | 0.3 (-0.9, 1.6) |  | 0.2 (-1.0, 1.4) |  |
|  |  |  |  |  |  |
| GAD-7 | Linear term | 1.2 (0.1, 2.3) | 0.08 | 1.1 (0.0, 2.1) | **0.01** |
|  | Squared term | -0.04 (-0.08, -0.01) |  | -0.04 (-0.08, -0.01) |  |
|  |  |  |  |  |  |
| sHAI ^(*)^ | Linear term | -8.8 (-18.8, 1.2) | **0.002** | -9.4 (-18.8, -0.1) | **<0.001** |
|  | Squared term | 2.1 (0.0, 4.3) |  | 2.2 (0.2, 4.3) |  |
|  | Cubic term | -0.2 (-0.3, 0.0) |  | -0.2 (-0.3, 0.0) |  |
|  |  |  |  |  |  |
| PHQ-9 ^(*)^ | - | -0.2 (-0.8, 0.4) | 0.46 | -0.7 (-1.3, -0.1) | **0.02** |
|  |  |  |  |  |  |
| OCI-R ^(**)^ | - | 0.1 (-0.3, 0.5) | 0.73 | -0.2 (-0.7, 1.9) | 0.27 |
|  |  |  |  |  |  |
| AUDIT-C | - | 0.0 (-0.3, 0.2) | 0.78 | 0.0 (-0.2, 0.2) | 0.90 |
|  |  |  |  |  |  |

(*) Regression coefficients reported for a 5-unit increase in variable

(**) Regression coefficients reported for a 10-unit increase in variable

*Univariable associations with changes in CAS score from baseline to 6 months with changes in scores on clinical measures*

| Baseline score | Category / Term | Unadjusted CAS Baseline | | Adjusted CAS Baseline | |
| --- | --- | --- | --- | --- | --- |
|  |  | Coeff (95% CI) | P-value | Coeff (95% CI) | P-value |
|  |  |  |  |  |  |
| GAD-7 reduction | - | 0.4 (0.2, 0.5) | **<0.001** | 0.3 (0.2, 4.6) | **<0.001** |
|  |  |  |  |  |  |
| sHAI reduction ^(*)^ | - | 1.2 (0.7, 1.6) | **<0.001** | 1.1 (0.6, 1.5) | **<0.001** |
|  |  |  |  |  |  |
| PHQ-9 reduction ^(*)^ | Linear term | 0.9 (0.4, 1.5) | **<0.001** | 0.8 (0.3, 1.4) | **<0.001** |
|  | Squared term | 0.4 (0.2, 0.7) |  | 0.4 (0.1, 0.6) |  |
|  |  |  |  |  |  |
| OCI-R reduction ^(**)^ | - | 1.0 (0.5, 1.5) | **<0.001** | 1.0 (0.6, 1.5) | **<0.001** |
|  |  |  |  |  |  |
| AUDIT-C change | - | -0.2 (-0.6, 0.2) | 0.28 | -0.2 (-0.6, 0.2) | 0.31 |
|  |  |  |  |  |  |
| Vaccinated by 6 months ^(+)^ | No | 0 | 0.65 | 0 | 0.88 |
|  | Yes | -0.5 (-2.8, 1.8) |  | -0.2 (-2.4, 2.0) |  |
|  |  |  |  |  |  |

(*) Regression coefficients reported for a 5-unit increase in variable

(**) Regression coefficients reported for a 10-unit increase in variable

(+) Analysis performed on 132 patients due to missing vaccination status data for remaining patients
